# Supplementary material for: Global RNA editome landscape discovers reduced RNA editing in glioma: loss of editing of gamma-amino butyric acid receptor alpha subunit 3 (GABRA3) favors glioma migration and invasion
Source: PeerJ. 2020 Sep 29;8:e9755. doi: 10.7717/peerj.9755 (PMC7531343; doi:10.7717/peerj.9755)
Supplement: Supplemental Information 1 [file peerj-08-9755-s001.zip › SupplementaryFigures/Supplementary figure legends.docx]

**Supplementary figure legends**

**Supplementary figure 1: Analysis pipeline for filtering out RNA editing.** Summary of different filtration criteria used to find out RNA editing events.

**Supplementary figure 2: Distribution of editing events across the genome for TCGA LGG tumors. A.** Distribution of total editing events in different regions of the genome: in Alu repeat, non-Alu repeat and non-repeat regions. **B.** Distribution of total editing events in different regions of the genome: in exonic, UTR, intergenic and intronic regions. **C.** Distribution of different types of editing events in different regions of the genome (Alu repeat, non-Alu repeat and non-repeat). **D.** Distribution of RNA editing events across different portions of the genome (exonic, intronic, intergenic and UTR). **E.** Cumulative distribution function of editing levels of Alu repeat, non-Alu repeat and non-repeat regions for TCGA LGG tumors. Significance testing was performed using Mann–Whitney U-test. Black color p value is between alu repeat regions *versus* non-alu repeat regions; red color p value is between alu repeat regions *versus* non-repeat regions.

**Supplementary figure 3: Distribution of editing events across the genome for TCGA GBM tumors. A.** Distribution of total editing events in different regions of the genome: in Alu repeat, non-Alu repeat and non-repeat regions. **B.** Distribution of total editing events in different regions of the genome: in exonic, UTR, intergenic and intronic regions. **C.** Distribution of different types of editing events in different regions of the genome (Alu repeat, non-Alu repeat and non-repeat). **D.** Distribution of RNA editing events across different portions of the genome (exonic, intronic, intergenic and UTR). **E.** Cumulative distribution function of editing levels of Alu repeat, non-Alu repeat and non-repeat regions for TCGA GBM tumors. Significance testing was performed using Mann–Whitney U-test. Black color p value is between alu repeat regions *versus* non-alu repeat regions; red color p value is between alu repeat regions *versus* non-repeat regions.

**Supplementary figure 4: Distribution of editing events across the genome for CGGA LGG tumors. A.** Distribution of total editing events in different regions of the genome: in Alu repeat, non-Alu repeat and non-repeat regions. **B.** Distribution of total editing events in different regions of the genome: in exonic, UTR, intergenic and intronic regions. **C.** Distribution of different types of editing events in different regions of the genome (Alu repeat, non-Alu repeat and non-repeat). **D.** Distribution of RNA editing events across different portions of the genome (exonic, intronic, intergenic and UTR). **E.** Cumulative distribution function of editing levels of Alu repeat, non-Alu repeat and non-repeat regions for TCGA CGGA tumors. Significance testing was performed using Mann–Whitney U-test. Black color p value is between alu repeat regions *versus* non-alu repeat regions; red color p value is between alu repeat regions *versus* non-repeat regions.

**Supplementary figure 5: Distribution of editing events across the genome for CGGA GBM tumors. A.** Distribution of total editing events in different regions of the genome: in Alu repeat, non-Alu repeat and non-repeat regions. **B.** Distribution of total editing events in different regions of the genome: in exonic, UTR, intergenic and intronic regions. **C.** Distribution of different types of editing events in different regions of the genome (Alu repeat, non-Alu repeat and non-repeat). **D.** Distribution of RNA editing events across different portions of the genome (exonic, intronic, intergenic and UTR). **E.** Cumulative distribution function of editing levels of Alu repeat, non-Alu repeat and non-repeat regions for CGGA GBM tumors. Significance testing was performed using Mann–Whitney U-test. Black color p value is between alu repeat regions *versus* non-alu repeat regions; red color p value is between alu repeat regions *versus* non-repeat regions.

**Supplementary figure 6: Distribution of editing events across the genome for CCLE glioma cell lines. A.** Distribution of total editing events in different regions of the genome: in Alu repeat, non-Alu repeat and non-repeat regions. **B.** Distribution of total editing events in different regions of the genome: in exonic, UTR, intergenic and intronic regions. **C.** Distribution of different types of editing events in different regions of the genome (Alu repeat, non-Alu repeat and non-repeat). **D.** Distribution of RNA editing events across different portions of the genome (exonic, intronic, intergenic and UTR). **E.** Cumulative distribution function of editing levels of Alu repeat, non-Alu repeat and non-repeat regions for CCLE glioma cell lines. Significance testing was performed using Mann–Whitney U-test. Black color p value is between alu repeat regions *versus* non-alu repeat regions; red color p value is between alu repeat regions *versus* non-repeat regions.

**Supplementary figure 7: Probability density distribution of RNA editing events.** Probability density distribution of RNA editing events of different regions of the genome (Alu repeat, non-Alu repeat and non-repeat) in control brain samples, TCGA LGG, TCGA GBM, CGGA LGG, CGGA GBM and CCLE glioma cell lines datasets.

**Supplementary figure 8: mRNA expression of ADAR family of enzymes. A.** Total number of RNA editing events in each dataset. (**B, C and D**). Scatter plots representing mRNA expression of ADAR, ADARB1 and ADARB2 in control brain samples, TCGA LGG (n=511), TCGA GBM (n=172), CGGA LGG (n=174), CGGA GBM (n=100) and CCLE glioma cell lines (n=45). Significance testing was performed using Mann–Whitney U-test.

**Supplementary figure 9: Differential RNA editing events in glioma. A.** Heat map representing two-way hierarchical clustering of significant differential RNA editing events in CGGA LGG (n=174) versus control brain (n=63) samples. Samples are shown vertically, magenta colour represents control brain and orange colour represents CGGA LGG tumors. RNA editing are shown in rows, magenta colour represents upregulated editing events in CGGA LGG tumors as compared to control brain samples and orange colour represents downregulated editing events in CGGA LGG tumors as compared to control brain samples. **B.** Heat map representing two-way hierarchical clustering of significant differential RNA editing events in CGGA GBM (n=100) versus Control brain (n=63) samples. Samples are shown vertically, magenta colour represents control brain and orange colour represents CGGA GBM tumors. RNA editing are shown in rows, magenta colour represents upregulated editing events in CGGA GBM tumors as compared to control brain samples and orange colour represents downregulated editing events in CGGA GBM tumors as compared to control brain samples. **C.** Heat map representing two-way hierarchical clustering of significant differential RNA editing events in CCLE glioma cell lines (n=45) versus Control brain (n=63) samples. Samples are shown vertically, magenta colour represents control brain and orange colour represents CCLE glioma cell lines tumors. RNA editing are shown in rows, magenta colour represents upregulated editing events in CCLE glioma cell lines as compared to control brain samples and orange colour represents downregulated editing events in CCLE glioma cell lines as compared to control brain samples.

**Supplementary figure 10: Mutagenesis of GABRA to introduce editing event and design of real time qPCR primers. A.** Sanger sequencing chromatogram to verify editing by site-directed mutagenesis. **B.**  RNA levels of GABRA3 in glioma samples compared to normal brain (NB) sample. **C.** To detect endogenous GABRA3 RNA levels, the forward primer was designed complementary to the 5’ UTR of the gene. To detect construct-specific GABRA3 RNA levels, the forward primer was designed complementary to the CMV promoter of the construct. The RNA level of GABRA3 was determined by real time qPCR. The plasmid GABRA3 RNA levels was normalised to the endogenous GABRA3 level for each condition.

**Supplementary figure 11: GABRA3 protein levels in T98G glioma cell line. A.** Protein levels of GABRA3 gene in T98G glioma cells having edited/unedited GABRA3 overexpressed ectopically in comparison to VC. **B.** Original blots with protein marker.

**Supplementary figure 12: GABRA3 protein levels in LN229 glioma cell line.** Original blots for protein level of GABRA3 gene in LN229 glioma cells having edited/unedited GABRA3 overexpressed ectopically in comparison to VC.
