# Supplementary figures and images for: Global RNA editome landscape discovers reduced RNA editing in glioma: loss of editing of gamma-amino butyric acid receptor alpha subunit 3 (GABRA3) favors glioma migration and invasion
Source: PeerJ. 2020 Sep 29;8:e9755. doi: 10.7717/peerj.9755 (PMC7531343; doi:10.7717/peerj.9755)

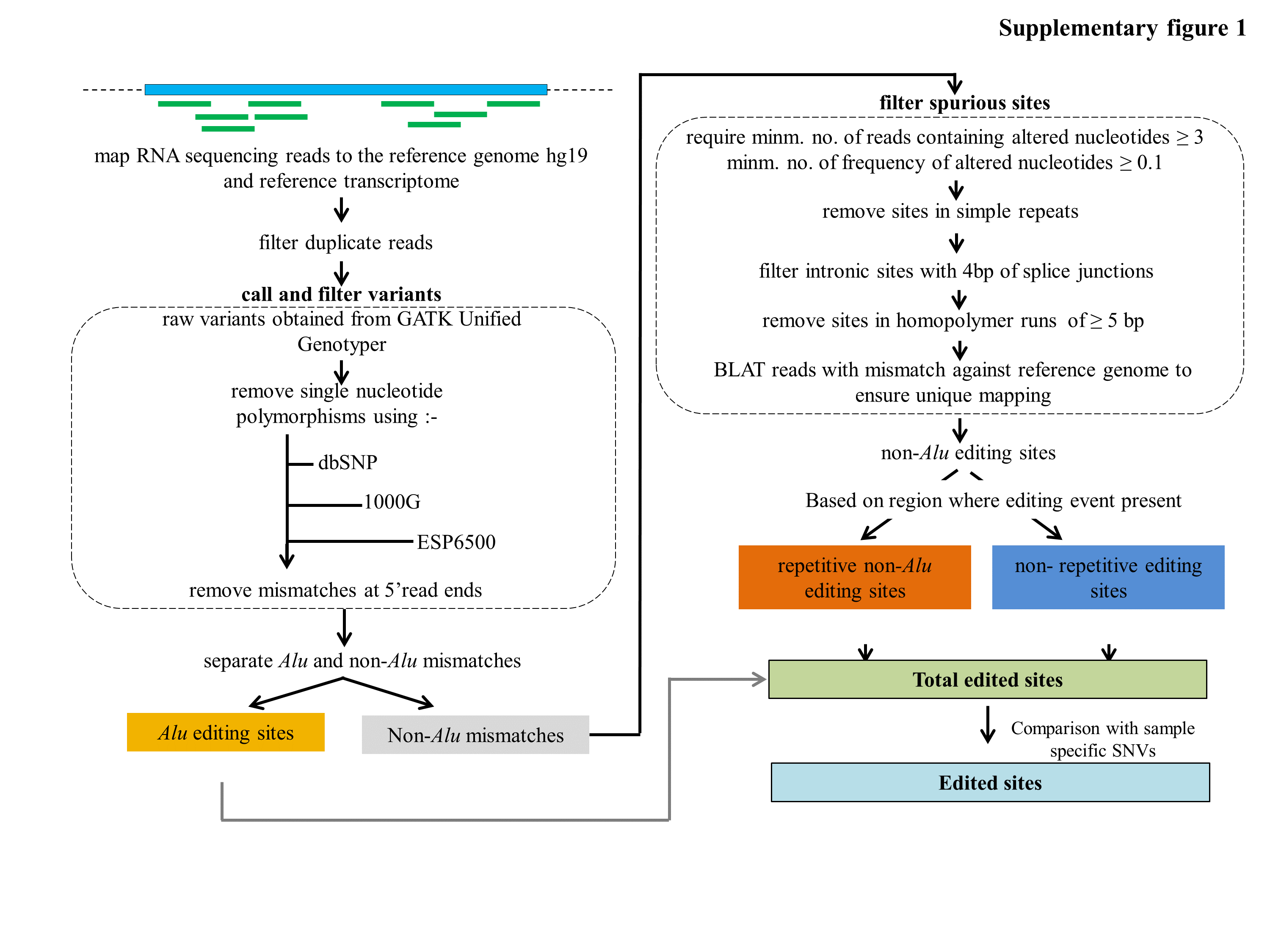

Supplement: Supplemental Information 1 [file peerj-08-9755-s001.zip › SupplementaryFigures/SupFig1.PNG]

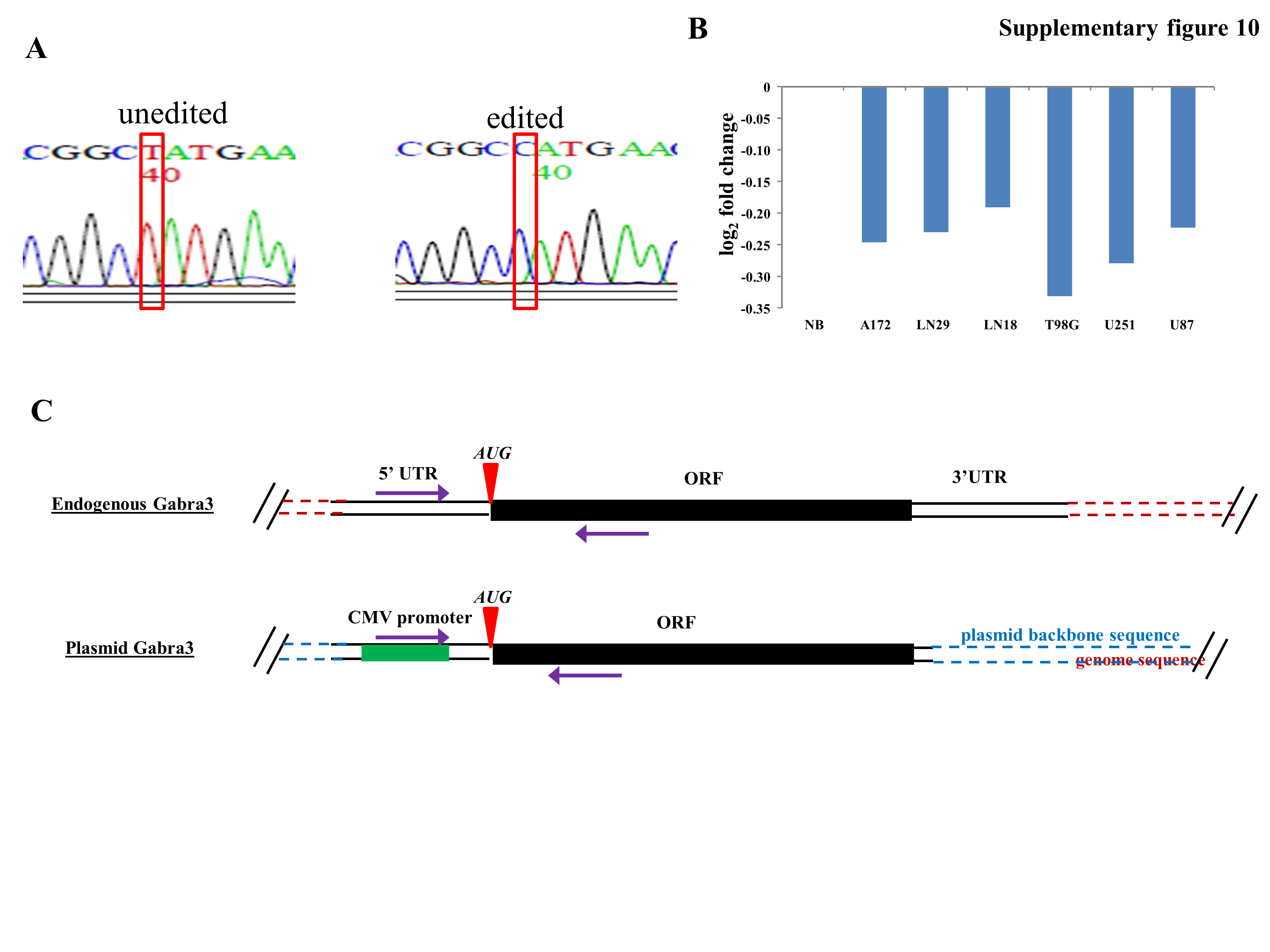

Supplement: Supplemental Information 1 [file peerj-08-9755-s001.zip › SupplementaryFigures/SupFig10.PNG]

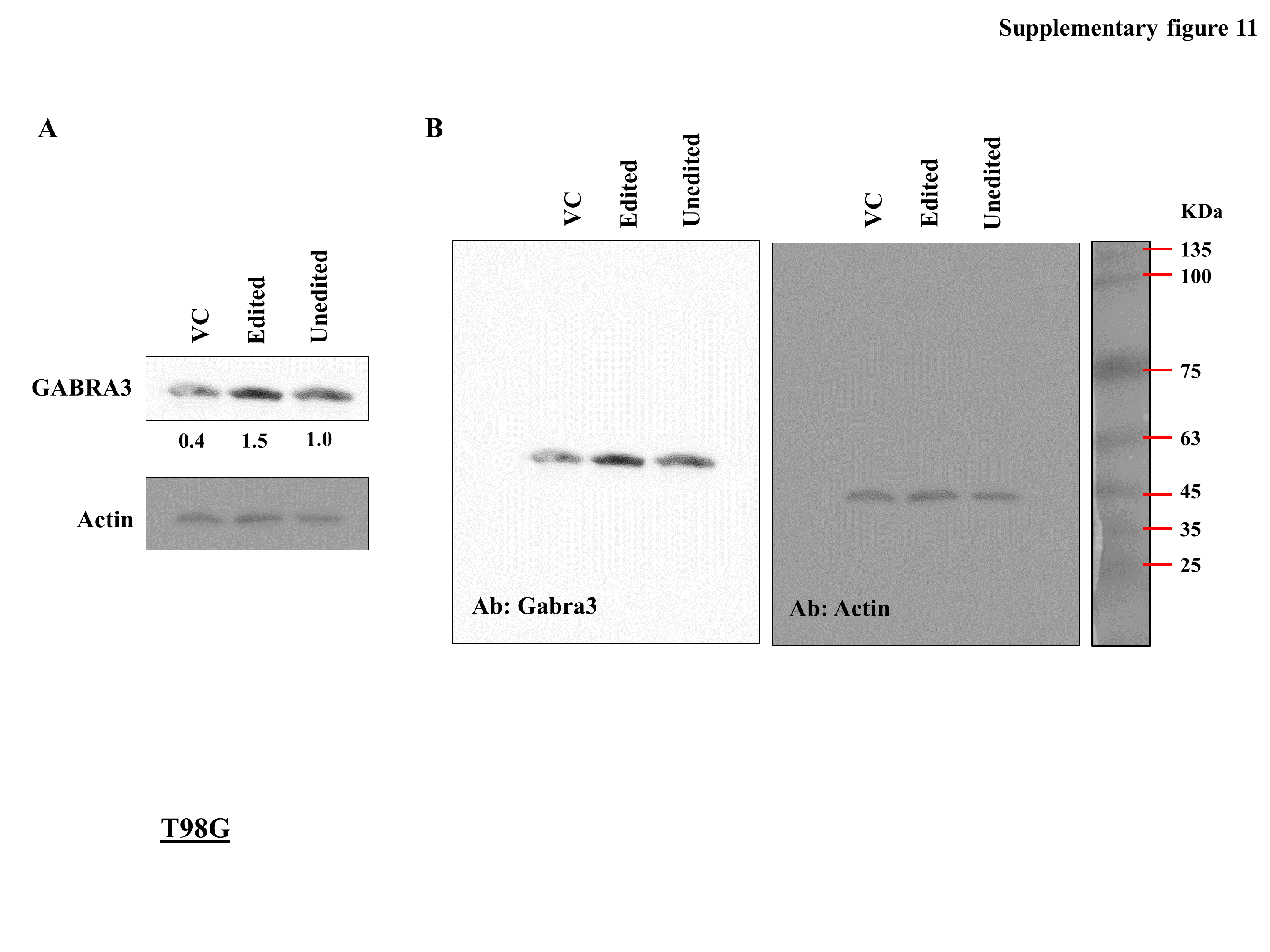

Supplement: Supplemental Information 1 [file peerj-08-9755-s001.zip › SupplementaryFigures/SupFig11.PNG]

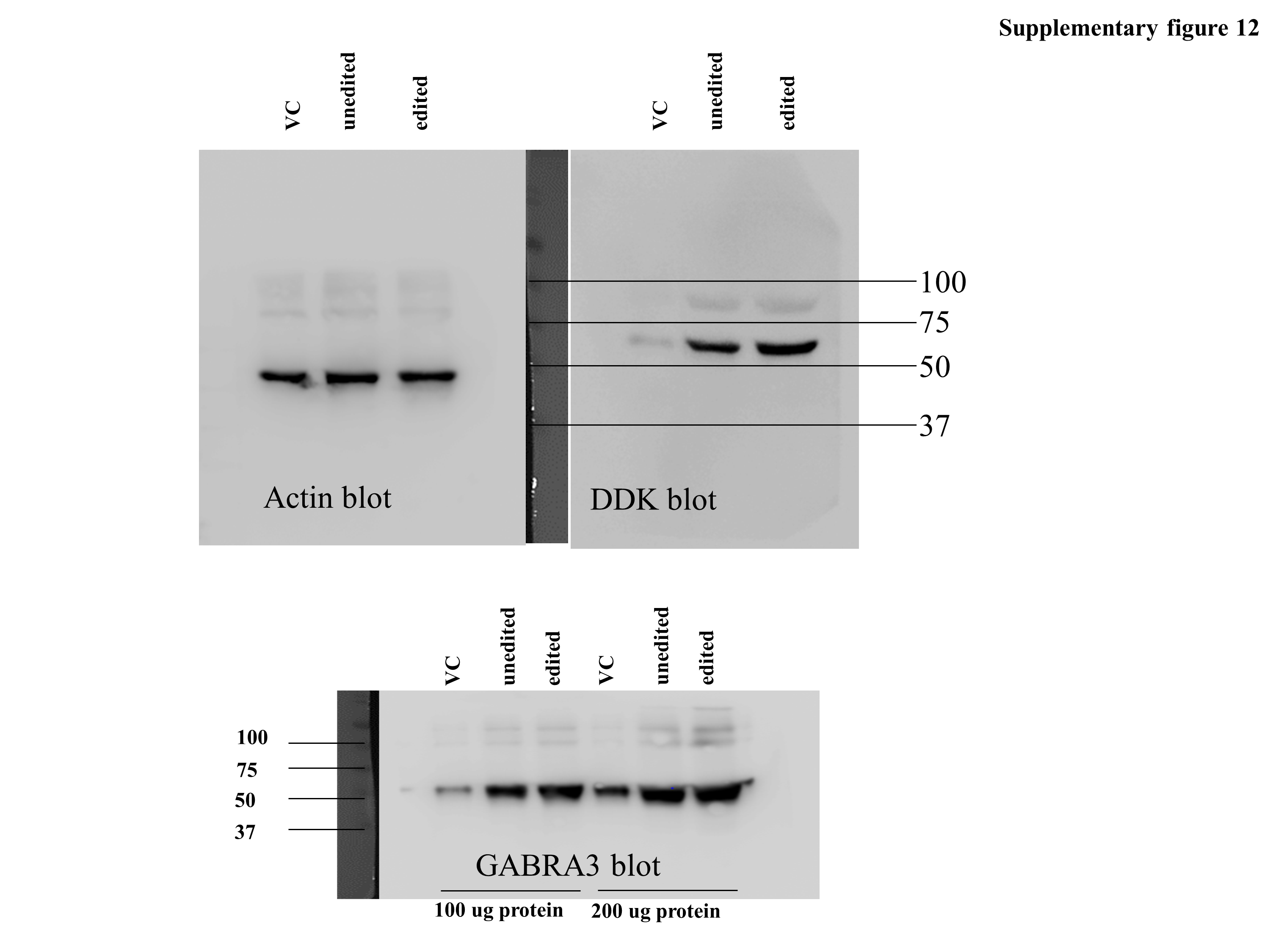

Supplement: Supplemental Information 1 [file peerj-08-9755-s001.zip › SupplementaryFigures/SupFig12.PNG]

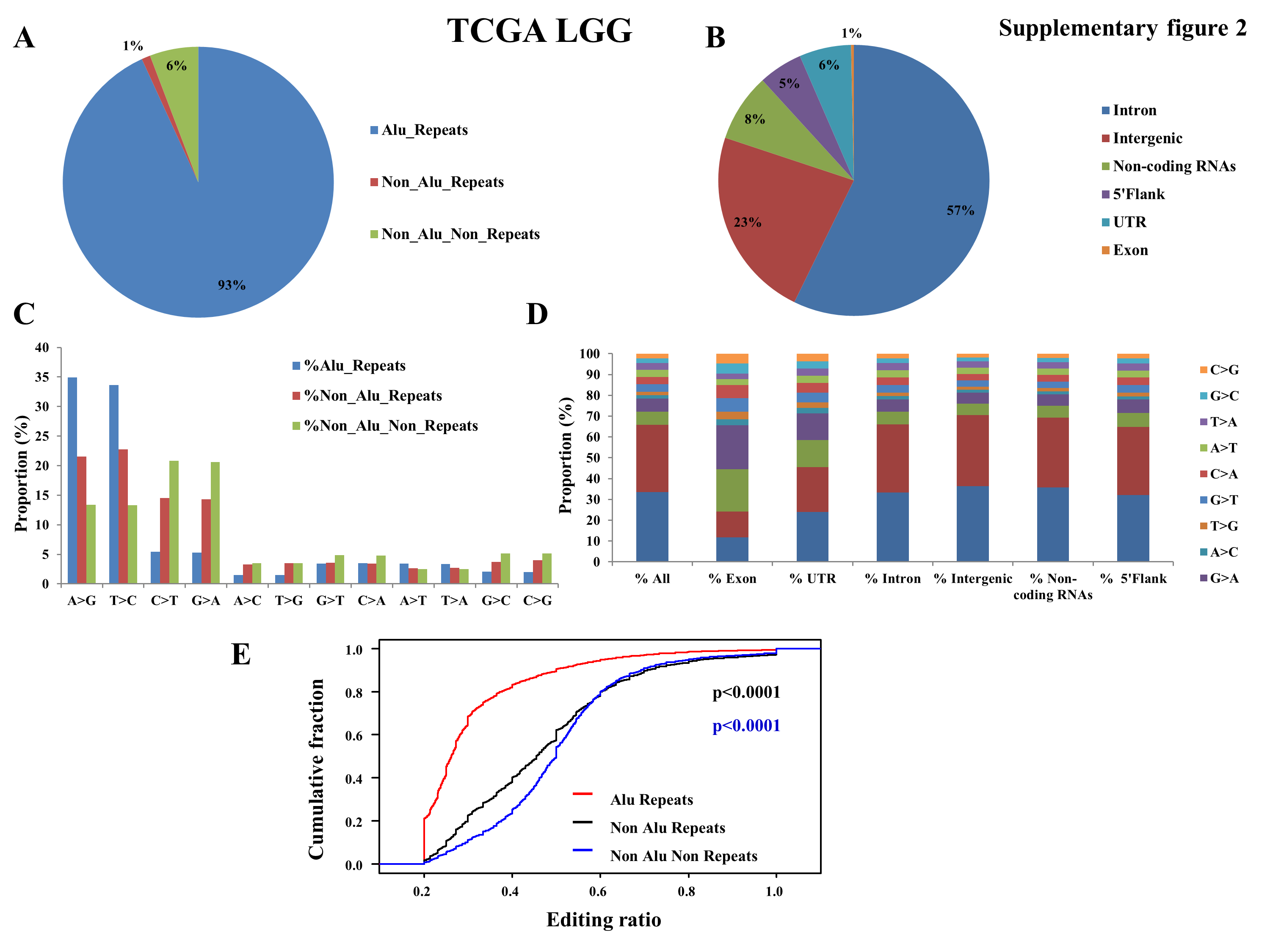

Supplement: Supplemental Information 1 [file peerj-08-9755-s001.zip › SupplementaryFigures/SupFig2.PNG]

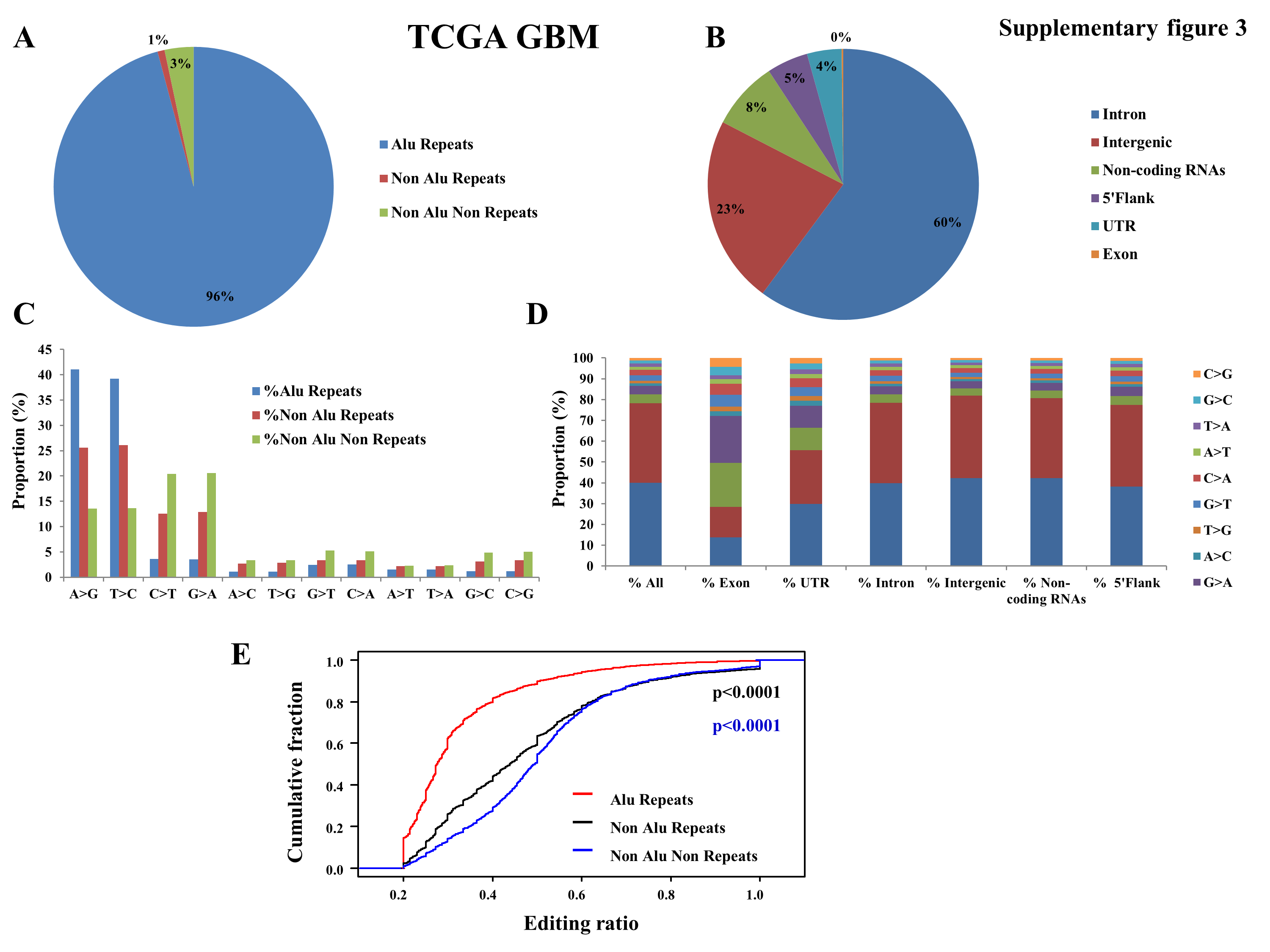

Supplement: Supplemental Information 1 [file peerj-08-9755-s001.zip › SupplementaryFigures/SupFig3.PNG]

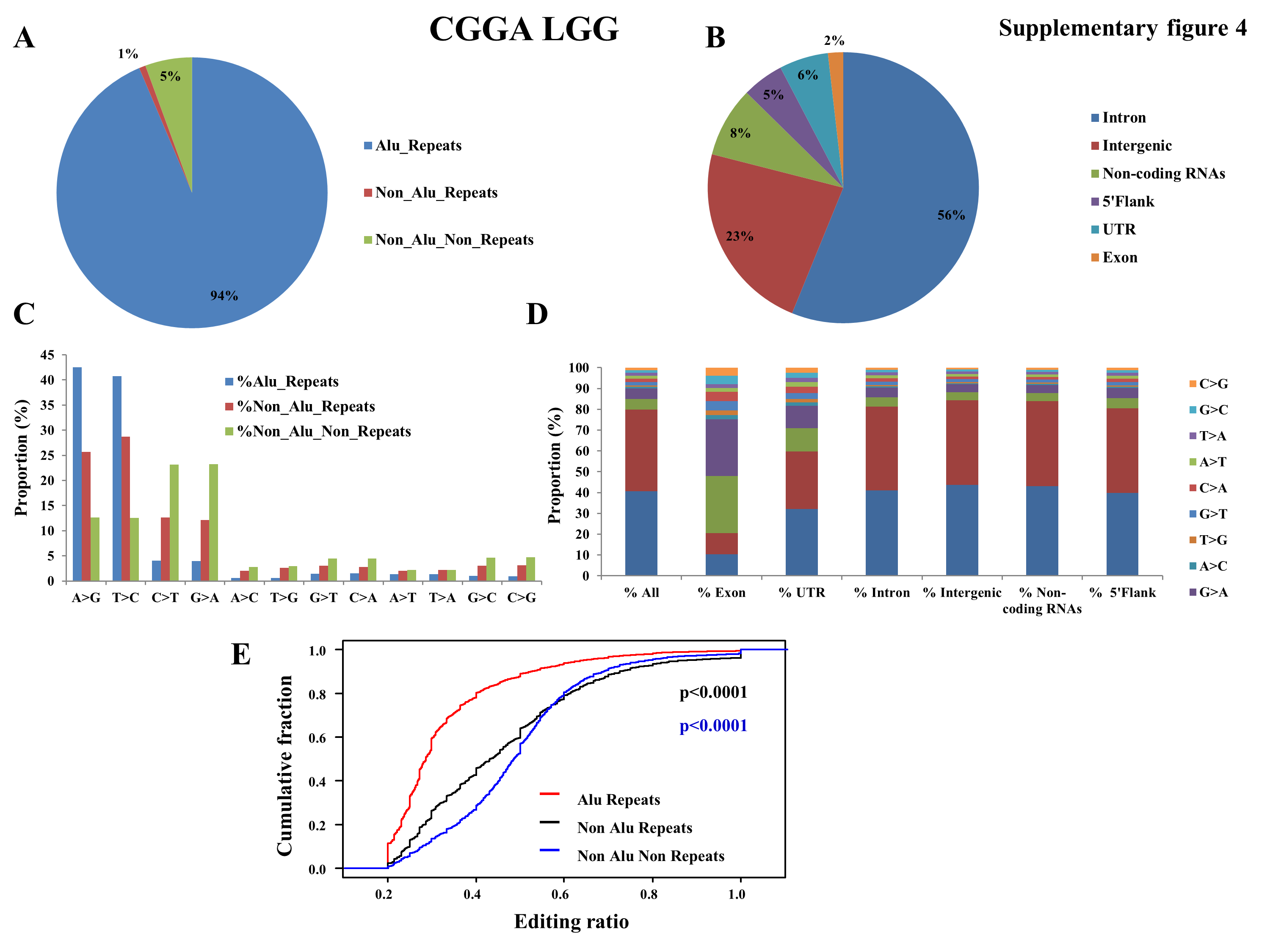

Supplement: Supplemental Information 1 [file peerj-08-9755-s001.zip › SupplementaryFigures/SupFig4.PNG]

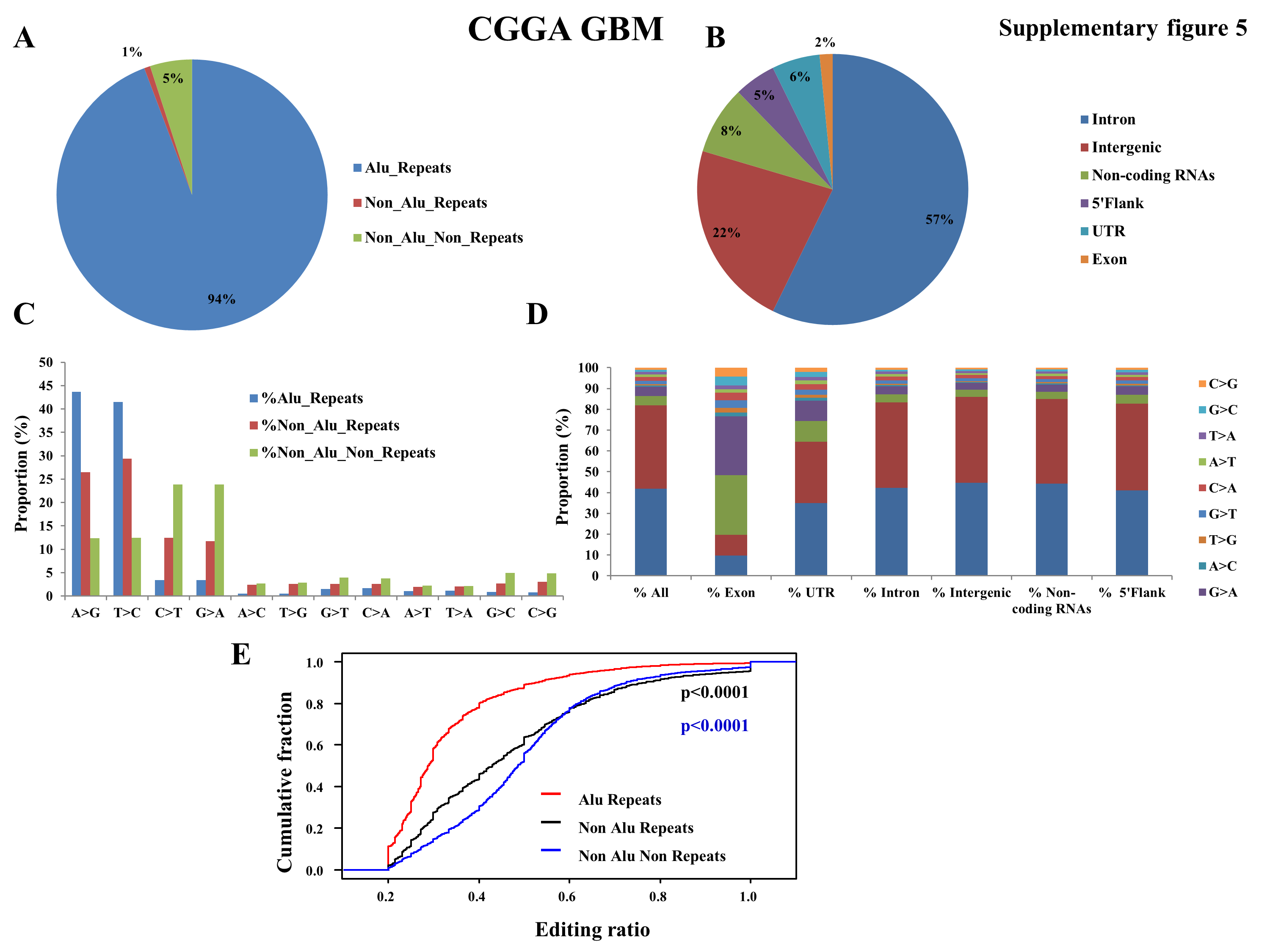

Supplement: Supplemental Information 1 [file peerj-08-9755-s001.zip › SupplementaryFigures/SupFig5.PNG]

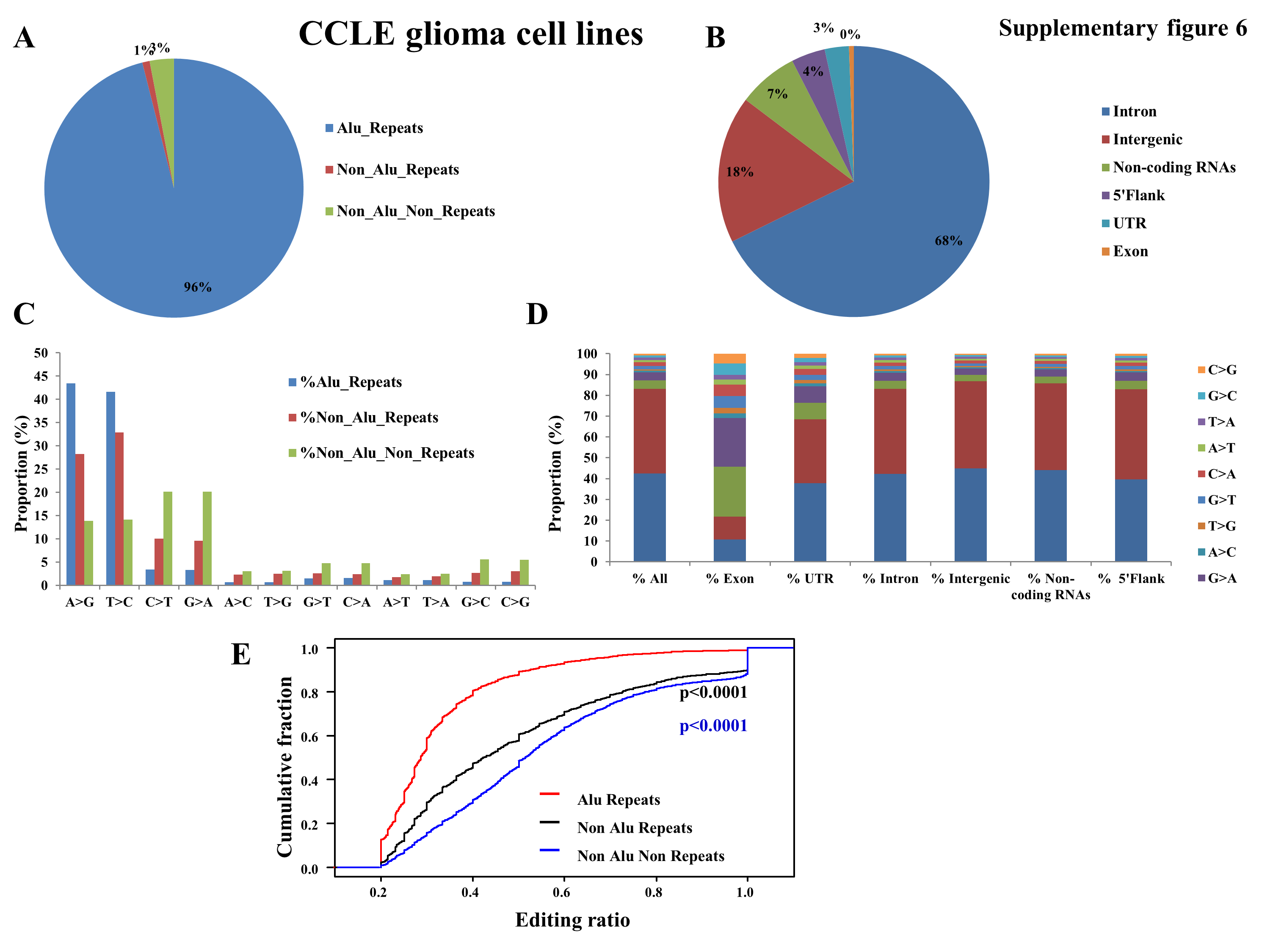

Supplement: Supplemental Information 1 [file peerj-08-9755-s001.zip › SupplementaryFigures/SupFig6.PNG]

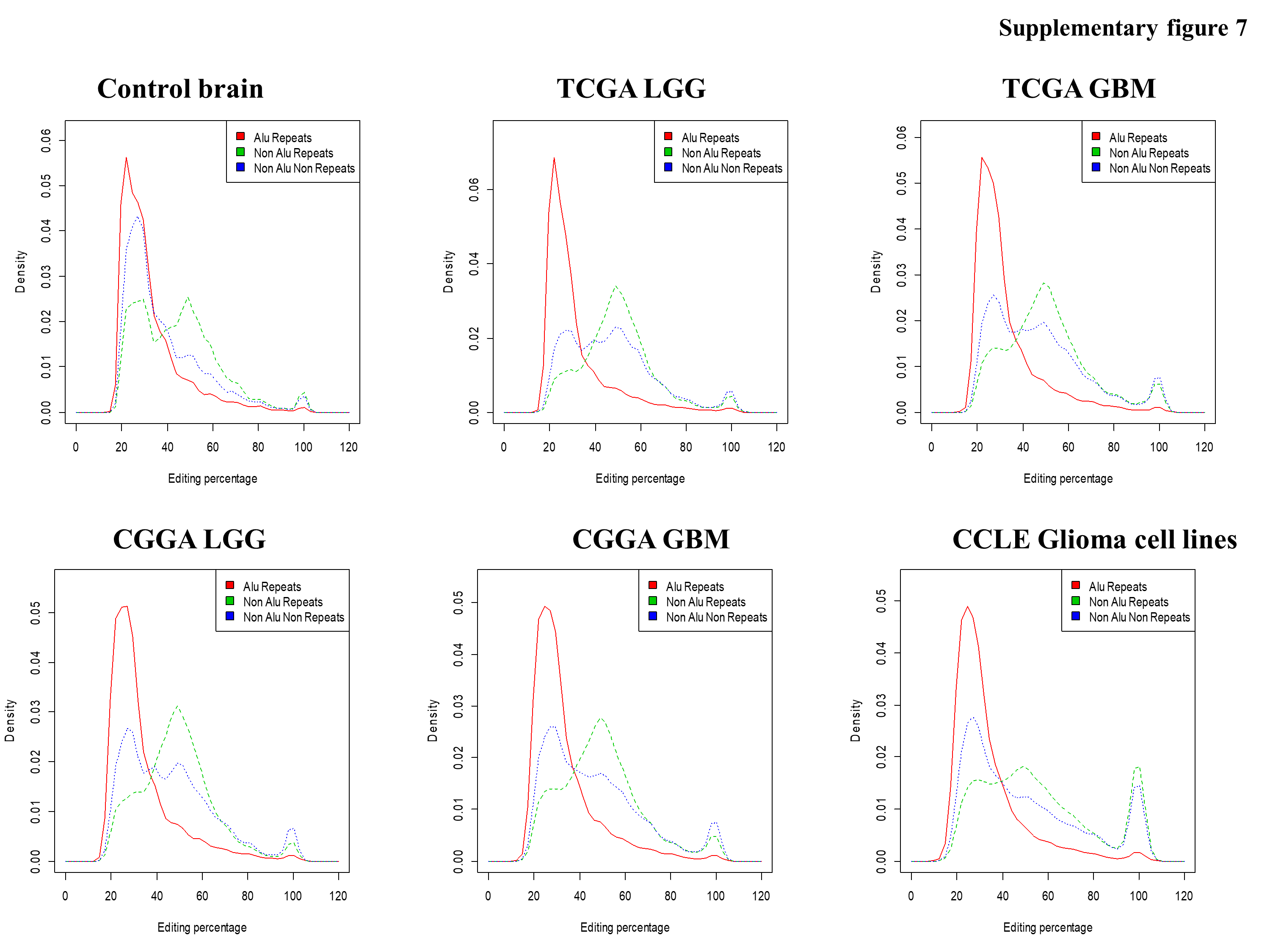

Supplement: Supplemental Information 1 [file peerj-08-9755-s001.zip › SupplementaryFigures/SupFig7.PNG]

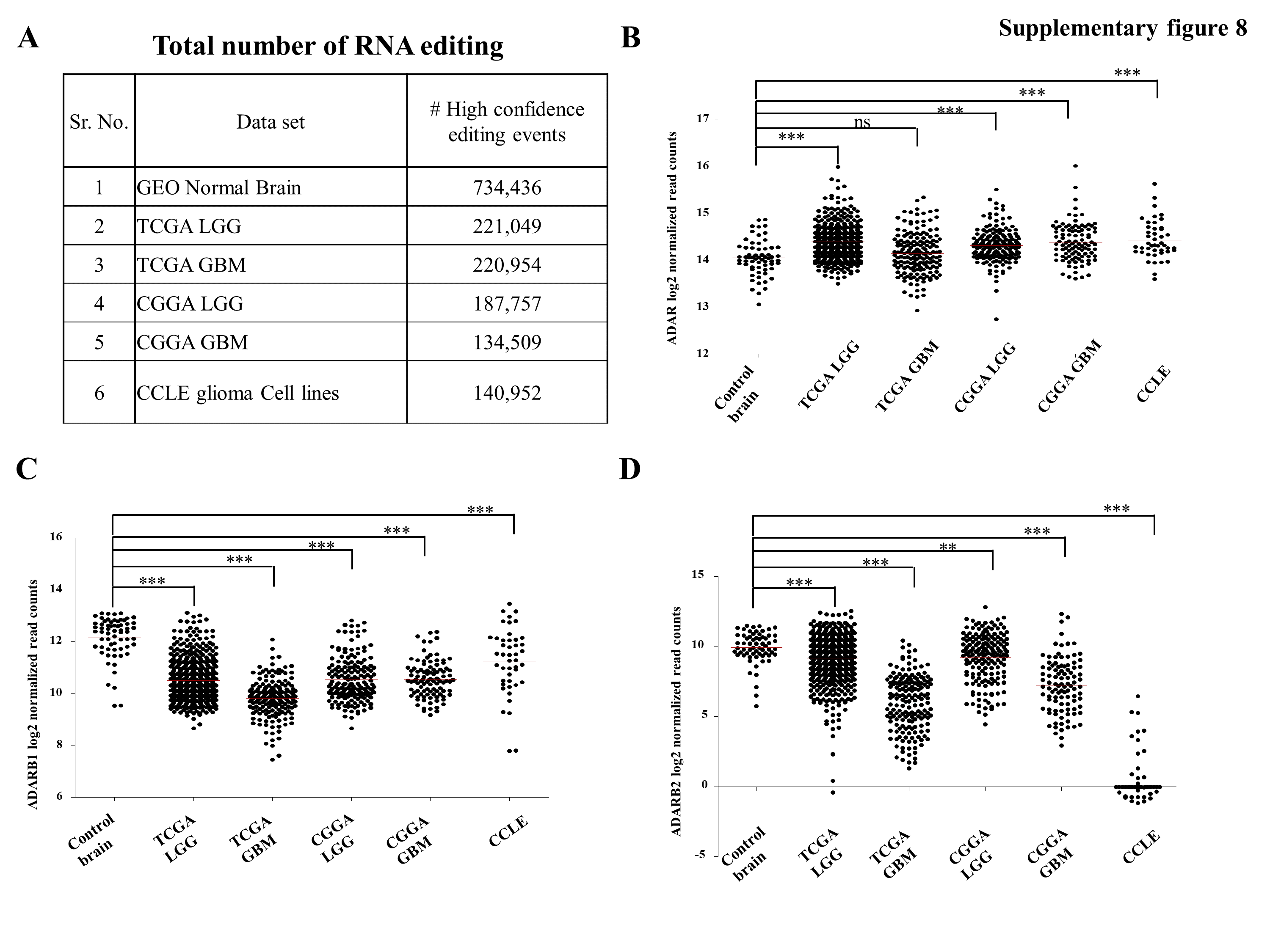

Supplement: Supplemental Information 1 [file peerj-08-9755-s001.zip › SupplementaryFigures/SupFig8.PNG]

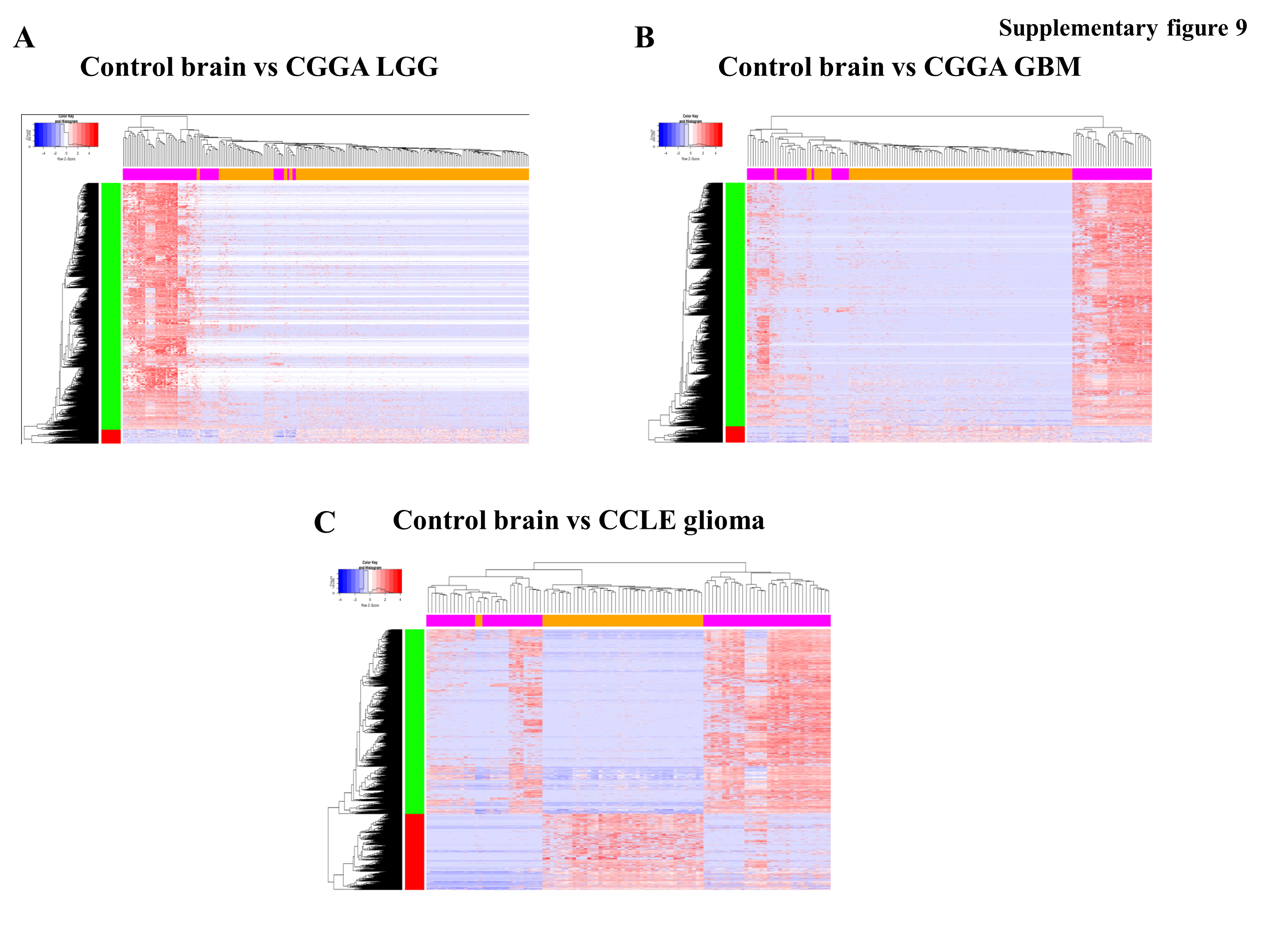

Supplement: Supplemental Information 1 [file peerj-08-9755-s001.zip › SupplementaryFigures/SupFig9.PNG]
